# Southern rice black-streaked dwarf virus (SRBSDV) directly affects the feeding and reproduction behavior of its vector, *Sogatella furcifera* (Horváth) (Hemiptera: Delphacidae)

**DOI:** 10.1186/1743-422X-11-55

**Published:** 2014-03-24

**Authors:** Hongxing Xu, Xiaochan He, Xusong Zheng, Yajun Yang, Junce Tian, Zhongxian Lu

**Affiliations:** 1 State Key Laboratory Breeding Base for Zhejiang Sustainable Pest and Disease Control, Institute of Plant Protection and Microbiology, Zhejiang Academy of Agriculture Sciences, Add. No 198 Shiqiao Rd, Hangzhou 310021, P R China; 2 Jinhua Academy of Agricultural Sciences, Jinhua 321017, P R China

**Keywords:** Southern rice black-streaked dwarf virus (SRBSDV), Whitebacked planthopper (WBPH), *Sogatella furcifera*, Feeding behavior, Reproduction

## Abstract

**Background:**

Southern rice black-streaked dwarf virus (SRBSDV) is a recently discovered member of the genus *Fijivirus* and it is transmitted by the rice whitebacked planthopper (WBPH), *Sogatella furcifera* (Horváth). It was found that SRBSDV infected vectors might contribute negatively to the WBPH population, although the longer nymphal period might benefit viral acquisition, transmission and increase infection rate. The interaction between SRBSDV and its vector need to be further explored to gain better understanding of the dispersal of WBPH and the spread of virus disease, in particular the feeding and reproduction behavior of viruliferous WBPH.

**Methods:**

Newly hatched nymphs of WBPH were fed on healthy rice plant after feeding on SRBSDV-infected rice plants for 2 h, and newly emerged adults were numbered and tested. Feeding behaviors of WBPH adults were monitored electronically within a Faraday cage using a Giga-4 DC EPG amplifier. The newly emerged adults were paired, and the fecundity and egg hatchability were investigated. WBPH was molecularly identified for SRBSDV when they dead. According to the identification results, data on viruliferous and non-viruliferous WBPH were collected and analyzed.

**Results:**

Feeding behavior of viruliferous WBPH was different from those of non-viruliferous WBPH. Frequency of phloem sap ingestion of viruliferous WBPH increased significantly, however the total feeding duration did not increase markedly. When both WBPH parents were infected with SRBSDV, their fecundity and hatchability of the eggs produced were significant lower than those of normal WBPH parents. However, if only one of the parents was viruliferous, fecundity and egg hatchability were only slightly affected.

**Conclusions:**

Viruliferous WBPH fed on the phloem more frequently than non-viruliferous WBPH and can thus contribute to virus transmission. When both vector parents are viruliferous fecundity and hatchability of the eggs were significantly reduced. However when only one of the parents WBPH was viruliferous, there were no significant effects.

## 

*Southern rice black-streaked dwarf virus* (SRBSDV) transmitted by the whitebacked planthopper (WBPH), *Sogatella furcifera* (Horváth), is a recently identified member of the genus *Fijivirus* in the family Reoviridae. The virus disease has caused disastrous damages in rice and maize production in China [1,2]. It was also recently recorded in Vietnam [3,4] and Japan [5]. In 2010, more than 60,000 ha of paddy fields in 29 provinces of Vietnam and more than 1,300,000 ha in 13 provinces of China were infected [3,4,6] while in 2011 the SRBSDV damaged more than 700,000 ha and in 2012 over 500,000 ha in China and Vietnam [7]. The losses in rice production can threaten food safety in these areas [3,7].

Since it was discovered, some studies had been conducted. Pu et al. (2012) showed that the frequency of viruliferous WBPH increased with the longer access time after 24 h virus feeding [8]. Viruliferous WBPH with SRBSDV feeding on viruliferous rice plant showed significant changes in fitness, including prolonged nymphal stages and reduced survival rates. Viruliferous rice plants also affected non-viruliferous WBPH by extending their survival rates at the temperature of 20°C and lowering their survival rates at a higher temperature of 28°C. Furthermore, both viruliferous WBPH and non-viruliferous WBPH populations had significantly shorter adult life spans at 25°C and lower fecundity at 28°C relative to the control [9]. In general, both SRBSDV infected vectors and host plants were unfavorable to WBPH population growth but longer nymphal periods [10]. A longer nymphal period might benefit viral acquisition and transmission by nymphs and might increase local infection rates [9]. Interactions between SRBSDV and its vector need to be further explored to better understand the dispersal of WBPH and the spread of virus.

There are generally different effects of viruses on the biology of vector insect [11,12]. Ecological fitness and feeding behavior of vectors may also be affected [13,14]. However, the differences in how the virus might affect the feeding and reproduction of viruliferous and non-viruliferous WBPH have not been explored.

In this paper we report studies on the feeding behavior of viruliferous and non-viruliferous WBPH. In addition we also assessed the effects on the reproduction charactersistics of WBPH when both or one of the parents infected with SRBSDV. The results would help to further understand the relationship between SRBSDV and its vector to improve the management of WBPH and the SRBSDV disease.

## Results

### EPG waveforms of WBPH

**Figure 1:**
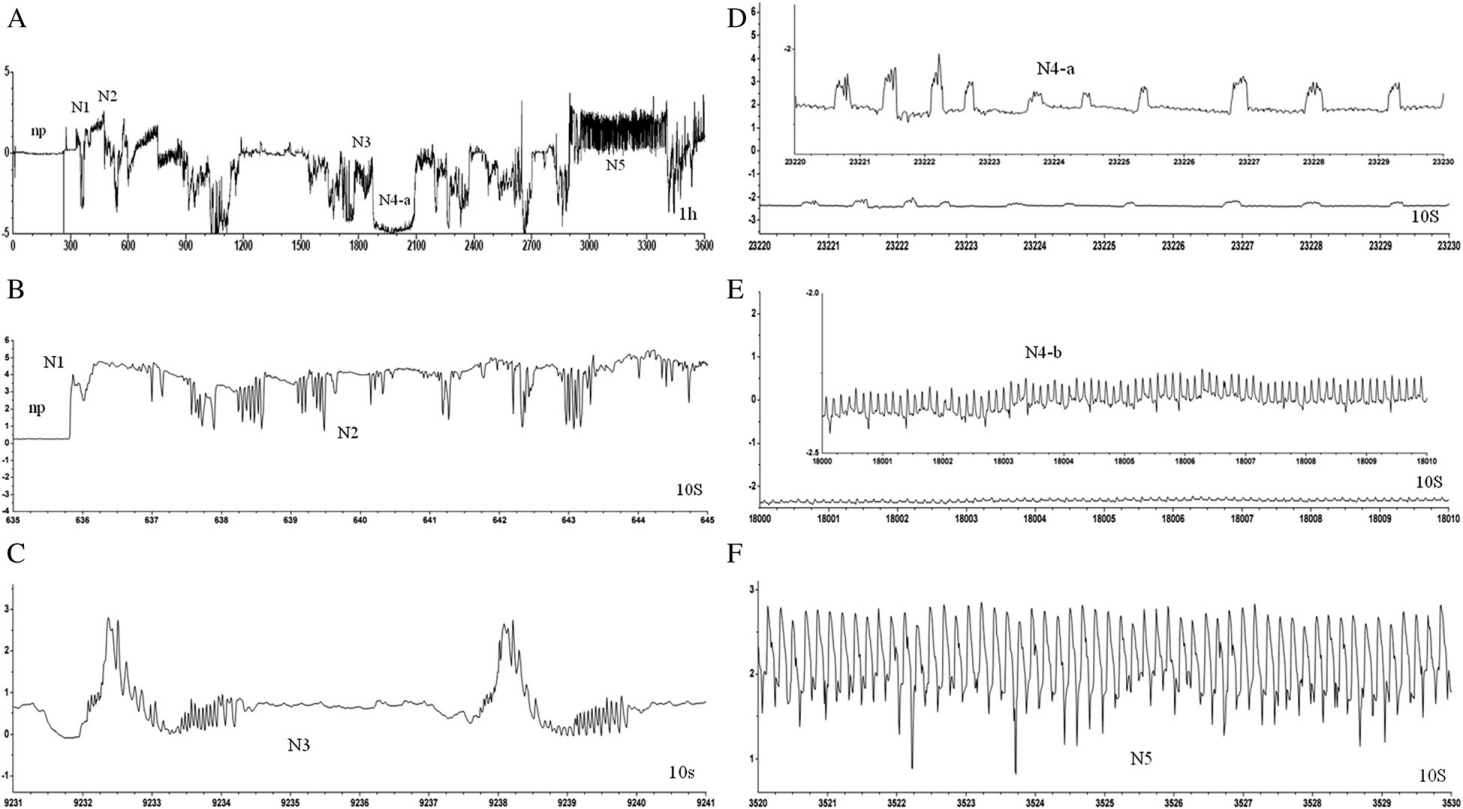
**Typical EPG waveforms recorded from viruliferous and non-viruliferous WBPH. (A)** The general picture during 1 h of recording; **(B-F)** the characteristic waveform in detail; np, non-penetration of stylets; N1, penetration initiation; N2, stylet movement; N3, an extracellular activity near the phloem region; N4 (N4-a and N4-b), phloem sap ingestion; N5, xylem sap ingestion.

The EPG waveforms recorded from viruliferous and non-viruliferous WBPH were similar. There were seven distinctive waveforms, non-penetration (np), penetration initiation (N1), salivation and stylet movement (N2), extracellular activity near the phloem region (N3), pathway phase (NC), intracellular activity in the phloem tissue (N4a), phloem sap ingestion (N4b), stylets in the phloem tissue (N4) (including N4-a and N4-b), and stylets in the xylem tissue (N5) (Figure 1).

The parameters of EPG waveforms recorded from viruliferous and non-viruliferous WBPH are shown in Table 1. Duration of non-penetration of stylets, total durations of N4-a and N5 waveforms of viruliferous WBPH were prolonged, and the numbers of occurrences of N1, N2, N3, N4 and N5 waveforms were also higher than those of non-viruliferous WBPH. However, the differences were not significant. The number of N4-b waveform occurrence in the viruliferous WBPH was obviously higher, and the average and total duration of N4-b waveform were shorter than those of non-viruliferous WBPH. This indicated that viruliferous WBPH fed in phloem more frequently than the non-viruliferous WBPH. On the other hand the frequency and duration of xylem sap ingestion (N5 waveform) of viruliferous WBPH fed in phloem were slightly higher and longer than those of non-viruliferous WBPH.

**Table 1:** Parameters of EPG waveforms of female WBPH adult

**Parameters**	**Non-viruliferous**	**Viruliferous**	***P***
Number of occurrences of np	15.50 ± 2.23	24.13 ± 6.08	0.216
Average duration of np (min)	3.50 ± 0.51	4.07 ± 1.09	0.609
Total duration of np (min)	57.00 ± 11.54	75.80 ± 18.76	0.377
Number of occurrences of N1	15.42 ± 2.22	24.13 ± 6.08	0.212
Number of occurrences of N2	34.33 ± 4.66	40.00 ± 10.60	0.636
Number of occurrences of N3	7.92 ± 1.69	12.50 ± 6.41	0.421
Average duration of Nc (min)	4.80 ± 2.18	3.66 ± 0.80	0.686
Total duration of Nc (min)	105.62 ± 23.06	130.16 ± 33.99	0.542
Number of occurrences of N4-a	2.75 ± 1.00	4.25 ± 1.42	0.386
Average duration of N4-a (min)	5.37 ± 1.30	4.41 ± 2.31	0.699
Total duration of N4-a (min)	14.86 ± 4.00	23.84 ± 13.88	0.472
Number of occurrences of N4-b	0.83 ± 0.21	1.75 ± 0.37	0.030
Average duration of N4-b (min)	113.44 ± 29.41	53.99 ± 27.40	0.179
Total duration of N4-b (min)	126.71 ± 29.38	82.71 ± 32.37	0.338
Number of occurrences of N4	2.75 ± 1.00	4.63 ± 1.40	0.277
Total duration of N4 (min)	141.57 ± 31.27	106.55 ± 33.39	0.467
Number of occurrences of N5	13.92 ± 3.99	16.00 ± 5.89	0.764
Average duration of N5 (min)	6.91 ± 1.58	15.14 ± 5.61	0.195
Total duration of N5 (min)	55.46 ± 11.73	96.31 ± 28.00	0.145
Percentage of Nc (N2 + N3) in the whole recording time	15.83 ± 3.21	21.06 ± 5.21	0.418
Percentage of np in the whole recording time	29.34 ± 6.40	36.16 ± 9.44	0.550
Percentage of N5 in the whole recording time	15.41 ± 3.26	26.75 ± 7.78	0.111
Percentage of N4 in the whole recording time	39.32 ± 8.69	29.60 ± 9.27	0.577
Percentage of N4-a in the whole recording time	4.13 ± 1.11	6.62 ± 3.86	0.739
Percentage of N4-b in the whole recording time	35.20 ± 8.16	22.98 ± 8.99	0.492

Note: Nc = N2 + N3; N4 = N4a + N4b. Data were means ± SE.

### Effects of SRBSDV on fecundity and egg hatchability of WBPH

Compared to control (♀_(−)_ × ♂_(−)_), the fecundity of viruliferous pairs of male and females were significantly decreased by 76.25%. The hatchability of eggs from these pairs was also reduced by 30.72%. When only one of the pairs was viruliferous fecundity and egg hatchability were slightly affected and not significantly different from the control (Table 2).

**Table 2:** Effects of SRBSDV on the fecundity, and egg hatchability of WBPH

**Combination**	**Number**	**Eggs laid/♀**	**Hatchability (%)**
♀_(+)_ × ♂_(−)_	15	56.33 ± 8.19ab	94.35 ± 0.04a
♀_(−)_ × ♂_(+)_	18	59.25 ± 15.73ab	93.53 ± 0.03a
♀_(+)_ × ♂_(+)_	10	16.50 ± 4.03b	66.67 ± 0.21b
♀_(−)_ × ♂_(−) (control)_	30	69.47 ± 6.88a	96.23 ± 0.02a

Note: ♀_(+)_ and ♂_(+)_ are female and male of WBPH infected with SRBSDV; ♀_(−)_ and ♂_(−)_ are female and male of WBPH uninfected with SRBSDV, respectively. Data were means ± SE. The data in the same column followed by different letters indicated the significant difference (*P* < 0.05).

## Discussion

Feeding behavior of the viruliferous vector may be altered by the plant virus. For instance the virus particles of *Maize streak virus* (MSV) were found in cells of phloem parenchyma, vascular bundle sheath and mesophyll tissue, and also in epidermal guard cells of plants infected with the maize strain of MSV. When the vector leafhopper *Cicadulina mbila* fed on MSV-infected plants, its feeding preferences was altered and the duration of feeding in mesophyll cell was prolonged. On healthy plants they primarily fed on the phloem. The feeding behavior changes benefited MSV by improving the acquisition and transmission efficiency of the vector [15]. Feeding behavior of vector beet leafhopper, *Circulifer tenellus* (Baker), was also found to be related with the transmission of *Beet severe curly top virus* (BSCTV). Waveform D1, which was associated with phloem salivation, was the only waveform correlated with inoculation of BSCTV [16]. Our results showed that the number of occurrence of N4-b waveform of viruliferous WBPH was obviously higher than that of non-viruliferous WBPH, and the average duration of N4-b waveform was shorter than that of non-viruliferous WBPH. It indicated that viruliferous WBPH fed in phloem more frequently than non-viruliferous WBPH and thus may benefit the spread of SRBSDV. It was also reported that both the viruliferous and non-viruliferous WBPH or *Rice ragged stunt virus* (RRSV)-carrying BPH preferred healthy plants to infected plants which might mean that plant viruses may alter host selection preference of vectors to enhance their spread and that of insects vectoring another virus to result in co-infection with more than one virus [10]. However, the specific stylet penetration behavior associated with inoculation of SRBSDV needs further investigation.

Our earlier studies showed that the fitness of viruliferous WBPH was affected. The nymphal duration of the male was prolonged markedly, the weight and fecundity of the female decreased significantly and the average survival time was shortened at both test temeperatures of 26°C and 31°C (Xu et al., unpublished data). It was reported that the viruliferous WBPH laid significant less eggs than non-viruliferous hoppers. There were no significant differences in the hatchability of eggs laid by virulifierous and non viruliferous females [9]. This study using paired viruliferous and nonviruliferous WBPH showed that both infected females and males had significantly reduced fecundity and the F1 egg hatchability. When paired with either a non viruliferous female or male, there were no significant effects in fecundity and egg hatchability. One may speculate that the genes involved in primary metabolism, ubiquitin-proteasome, cytoskeleton dynamics and immune responses were up regulated in viruliferous WBPH [17]. Since WBPH adult has active mobility and frequently move among rice plants, it is likely that pairing with only one viruliferous adult is more common and thus might not reduce WBPH populations. One viruliferous WBPH could transmit virus to 15 to 17 rice plants in five days or about 49 plants during its whole lifespan [8,18], and thus virus spread will probably be retained or increased [19].

Viriliferous vectors have reduced fecundities [20,21]. *Tomato yellow leaf curl virus* (TYLCV) infested vectors *Bemisia tabaci* (Genadius) also have about for 17-23% shorter longevity and reduced fecundity of about 40-50% [22]. Similarly in western flower thrips (WFT), *Frankliniella occidentalis* (Pergande), exposed to *Tomato spotted wilt virus* (TSWV) compared to the unexposed thrips reared on uninfected leaf discs, mean daily fecundity was reduced and the egg hatchability affected as well [23]. Parents of small brown planthopper (SBPH), *Laodelphax striatellus* (Fallen), especially the male, infected with *Rice striped virus* (RSV) also had significant effects on the egg hatchability of the next generation [24]. On the other hand some researchers have found that the total number of oocytes and the proportion of oocytes developed at the grade I status in the vector green leafhopper, *Nephotettix cincticeps* (Uhler), were significantly increased when they were fed on *Rice dwarf virus* (RDV)-infected rice plants [25].

Furthermore, Co-infection of the begomovirus *Tomato yellow leaf curl China virus* (TYLCCNV) and its betasatellite can repress a-regulated defences of tobacco against invasive whiteflies *Bemisia tabaci* (Gennadius) and accelerate population increases of the insects [26]. The tripartite interactions of plant–pathogen–vector relationships are complex. And these results provide an interesting model to study the plant–pathogen–vector interactions through an integration of ecological, physiological and molecular approaches.

## Conclusions

Our results showed viruliferous WBPH fed on phloem more frequently than non-viruliferous WBPH which might increase the probability of virus transmission. When only female or male WBPH was infected with SRBSDV, the fecundity and egg hatchability were lower than those of the non-infected pairs although not significant. If however both sexes were viruliferous, the fecundity and egg hatchability were adversely affected.

## Materials and methods

### Rice plants

Rice variety Y-liangyou1 was used in the experiment. It is a dominant *indica* hybrid rice in Wuyi county (119.81°E, 28.9°N), Zhejiang province, China, where the SRBSDV disease frequently outbreaks. Germinated rice seeds were sown at the rate of 15 kg per hectare and transplanted at one seedling per hill spaced 20 by 20 cm apart. A total of 90 kg N, 90 kg P and 144 kg K per hectare was applied as base fertilizer before planting. Another 90 kg N was applied in two splits at tillering stage (60%) and reproductive stage (40%). Rice was naturally infected by immigrant viruliferous WBPH. Thirty days after sowing, rice plants with visible SRBSDV-infected symptoms were marked. The healthy and infected rice plants with typical disease symptoms were uprooted and transplanted individually in clay pots (Φ = 15 cm). After all arthropods were manually eliminated, potted plants were covered with a cage made of a transparent plastic cylinder with nylon mesh at the top for ventilation. All plants were molecularly identified following the method of Li et al. (2012) [27].

### WBPH

A strain of WBPH were collected in experimental farm of China National Rice Research Institute in Hangzhou (119.95°E, 30.07°N), China and maintained on healthy rice plants in laboratory. Fifty 5th instar nymphs of 2nd generation were collected and reared individually. After emergence, male and female WBPH were paired and transferred to a caged rice plant for oviposition. Adults were identified by RT-PCR to confirm whether they were viruliferous or not after they were died. All offspring of virus-free of original insects were maintained on healthy rice plants at 26 ± 1°C with photoperiods of 12 L: 12D. Newly hatched nymphs of next generation were used in this study.

### Acquisition of viruliferous and non-viruliferous WBPH adults

Following the methods described by He *et al.* (2011) [28], 45-day-old healthy or SRBSDV-infected rice plants were removed outer sheaths and inactive roots and cleaned carefully with tap water and were placed in a glass tube (diameter 2.5 cm, height 18 cm) with 1.5 cm deep Kimura B nutrient solution individually. Ten newly hatched nymphs of WBPH were introduced into a glass tube with healthy rice plant after feeding on SRBSDV-infected rice plants for 2 h. All the glass tubes were kept in a climate chamber with 26 ± 1°C, relative humidity (RH) of 75-90% and photoperiods of 12 L: 12D. Rice plants were replaced and Kimura B nutrient solution was replenished if needed. When adults emerged, they were used in the following experiments.

### Effect of SRBSDV on feeding behaviors of WBPH adults

Following the methods described by He *et al.* (2011) [29], feeding behavior of WBPH adults were monitored electronically within a Faraday cage using a Giga-4 DC EPG amplifier with a 10^9^ Ω input resistance and an input bias current of less than 1 pA (Wageningen Agricultural University, The Netherlands). Female adults, which emerged within 8 h were transferred into a glass tube (diameter 2 cm, length 10 cm) and provided water only (water-saturated cotton) for two hours before used. One end of a gold wire (diameter 20 μm, length 10 cm) was attached to the dorsal thorax of WBPH with water-soluble silver conductive glue. The other end of the wire was connected to the amplifier through the EPG probe. A copper wire (diameter 2 mm, length 10 cm) was inserted into the soil to serve as the plant electrode. The female attached to the gold wire was carefully placed onto the stem of healthy rice plant. The gain of the amplifier was set at 50 ×, and the plant voltage was adjusted to obtain an output voltage of between −5 and +5 V. EPG signals were analyzed with the PROBE 3.0 software (Wageningen Agricultural University, The Netherlands). After EPG recording for 6 h, the females were identified for SRBSDV individually. According to the identification results, 22 and 18 replications were applied for viruliferous WBPH and non-viruliferous WBPH, respectively. All the EPG tests were conducted at 26 ± 1°C, 70 ± 5% RH and continuous light conditions.

EPG waveforms from WBPH recorded in the tests were characterized using the correlations found in Seo et al. (2009) [30] and He et al. (2011) [29]. Seven distinctive waveforms were classified and analyzed with a modified method as Sarria et al. (2009) described [31]. Four parameters of non-sequential variables of each EPG waveform, including total duration, average duration, number of occurrences and proportion of each waveform phase in whole recording time were analyzed.

### Effects of SRBSDV on reproduction of WBPH

Newly emerged adults were paired and transferred into a glass tube with two healthy rice tillers. They were allowed to mate and oviposit until death, and the fecundity was investigated as described below. Each tube was observed daily and newly hatched nymphs were counted and removed every day until no more nymphs hatched. Unhatched eggs were counted by dissecting under microscope. Death WBPH adults were collected and molecularly identified individually to confirm if they were infected with SRBSDV. According to the identification results, 15, 18, 10 and 30 replications were applied for the treatments viruliferous female ♀_(+)_ × non-viruliferous male ♂_(−)_, non-viruliferous female ♀_(−)_ × viruliferous male ♂_(+)_, ♀_(+)_ × ♂_(+)_ and ♀_(−)_ × ♂_(−)_, respectively.

### Statistical analysis

The data on feeding behavior of viruliferous and non-viruliferous WBPH were analyzed using student’s *t*-test. Single factor analysis of variance was conducted to analyze the fecundity and egg hatchability of WBPH (egg hatchability were transformed into arcsine and square root values, respectively before performing the statistical analyses). All statistical tests were performed using SPSS V18.0.

## Competing interests

The authors declare that they have no competing interests.

## Authors’ contributions

HX and XH carried out the experiments and drafted the manuscript. XZ was involved in collecting WBPH and SRBSDV-infected rice plants. YY and JT helped with experimental procedures and manuscript preparation. ZL designed the study and critically revised the manuscript. All authors read and approved the final manuscript.
